# The Benefits and Risks of Glucagon-Like Peptide-1 Receptor Agonists on Ocular Diseases: A Narrative Review

**DOI:** 10.7759/cureus.103234

**Published:** 2026-02-08

**Authors:** Jacky Xiao Feng Huang, Joshua Wu, Hunain Ahmad, Jason Li, Samaantar Joshi, Adil Yousaf, Rishita Pemminati, Sudhakar Pemminati

**Affiliations:** 1 Department of Biomedical Education, California Health Sciences University College of Osteopathic Medicine, Clovis, USA; 2 Department of Biology, California State University, Fresno, USA

**Keywords:** diabetic macular edema (dme), diabetic retinopathy, glucagon-like peptide-1 receptor agonists, non-arteritic anterior ischemic optic neuropathy (naion), retinal vascular occlusion, vitreous haemorrhage

## Abstract

Glucagon-like peptide-1 receptor agonists (GLP-1RAs) are incretin-based agents used in the management of type 2 diabetes mellitus (T2DM) and obesity. This class of medications mimics the action of the endogenous GLP-1 hormone, enhances insulin secretion, delays gastric emptying, causes early satiety, lowers systemic glycemic levels, and results in weight loss. There are emerging concerns about the use of GLP-1RAs, which may be an observational association with ocular pathologies, including retinal vascular occlusion (RVO), vitreous hemorrhage (VH), diabetic retinopathy (DR), non-arteritic ischemic optic neuropathy (NAION), and diabetic macular edema (DME). In contrast, there are beneficial impacts of GLP-1RAs on various conditions, including glaucoma and idiopathic intracranial hypertension. This narrative review aims to analyze current findings on the beneficial and adverse ocular effects of GLP-1RAs use, highlighting studies that elucidate the harmful causes, such as retinal microvascular dysregulation, inflammatory modulation, oxidative stress, osmotic dysregulation from sorbitol accumulation, and metabolic shifts. The literature search was conducted using PubMed, Elsevier, Embase, Google Scholar, Scopus, and Science Direct to retrieve relevant literature published in peer-reviewed journals from 2014 to 2025. Understanding the benefits and risks of GLP-1RAs in ophthalmology is crucial for clinicians to manage ocular conditions safely while a patient is using GLP-1RAs, and enables personalized ophthalmological screenings to minimize the risk of disease exacerbation.

## Introduction and background

Glucagon-like peptide 1 (GLP-1) is an incretin hormone secreted by intestinal L-cells that stimulates glucose-dependent insulin secretion and rapidly lowers glycemic levels. Given their well-studied metabolic effects, GLP-1 receptor agonists have emerged as effective therapies for blood glucose control and weight loss in patients with T2DM and obesity [1,2]. In 2024, more than one in four adults with diagnosed diabetes used GLP-1 injectables, about 26.5% [3]. GLP-1RAs demonstrate organ-spanning benefits, which range from anti-inflammatory effects in dermatological diseases, improvement in PCOS, nephroprotection, and reduction in cardiometabolic risk. Alternatively, GLP-1RAs present adverse effects in gastrointestinal intolerance and rare dermatological and neuropsychiatric adverse events [4]. The population of patients meeting clinical eligibility criteria for GLP-1s is one of the largest of any new drug class in the past 20-30 years. It is estimated that up to 5% of the US population could be prescribed a GLP-1 by 2030, for a variety of diseases, including cardiovascular and inflammatory disorders [5].

Recent clinical evidence has raised concerns regarding GLP-1 RAs use and its effects on ocular health in patients with pre-existing ocular diseases. Studies on GLP-1RA use have suggested adverse effects concerning diabetic macular edema (DME), vitreous hemorrhage (VH), retinal vascular occlusion (RVO), diabetic retinopathy (DR), and non-arteritic ischemic optic neuropathy (NAION) [6-8]. Conversely, emerging evidence also supports the protective roles of GLP-1RAs in other ocular conditions, such as primary open-angle glaucoma (POAG) and papilledema associated with idiopathic intracranial hypertension (IIH), through the reduction of intraocular and intracranial pressure, as well as neuroprotective mechanisms. 

This review aims to synthesize current clinical data regarding both the ocular risks and benefits of using GLP-1RAs and ultimately provides a comprehensive analysis that can help clinicians navigate ways to optimize use with individualized ocular disease screenings. This review supports safer use of GLP-1RAs for patients with ocular diseases.

Pathophysiology of GLP-1 RAs on ocular diseases**

**Figure 1:**
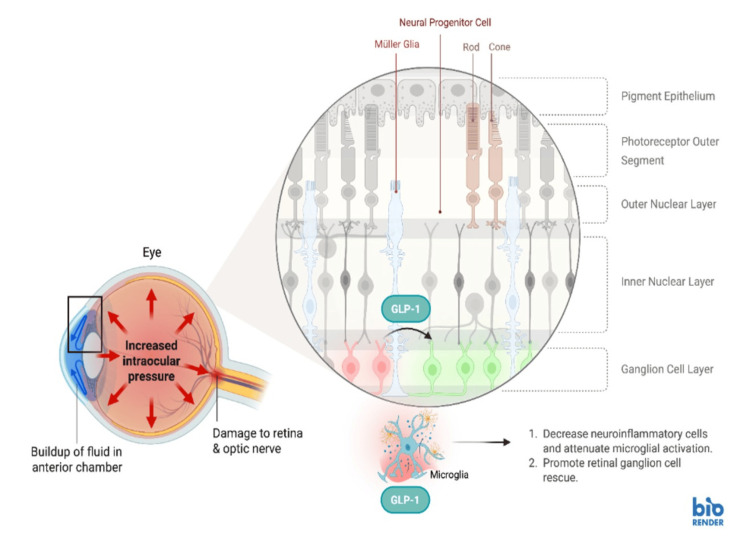
Effects of GLP-1RA on glaucoma Image Credit: Huang J. This image was created in BioRender (https://BioRender.com/00js6d7).

GLP-1 RAs exert both protective and adverse effects on the eye. This paradoxical interplay is mediated by changes in vascular regulation, inflammation, oxidative stress response, and rapid metabolic shifts that are a direct result of intensive glycemic control. On the protective side, GLP-1RAs improve retinal vascular regulation and help reduce inflammatory signaling in retinal tissues by preserving the blood-retinal barrier and lowering vascular permeability [9]. GLP-1RAs also have demonstrated neuroprotective traits by attenuating microglial activation and enhancing retinal ganglion cell survival [10]. Additionally, studies have further suggested that the use of GLP-1RAs can decrease the risk of ocular hypertension, primary open-angle glaucoma, and possibly improve intraocular pressure regulation through these protective mechanisms [11,12]. The protective effects of GLP-1RAs on glaucoma are illustrated in Figure 1.

Conversely, adverse ocular effects can arise from GLP-1RAs under certain conditions, mainly through vascular dysregulation secondary to metabolic shifts. These events are often reported in temporal proximity to rapid therapy initiation or dose escalation during the early phase of glycemic improvement when HbA1c falls most steeply, with the incidence of confirmed events highest when HbA1c falls >1.5% [13]. Rapid corrections in glycemic control, which is a hallmark metabolic effect of GLP-1 RAs, transiently worsen DR, in a pattern known as the “early worsening of diabetic retinopathy” (EWDR) phenomenon, which is described after abrupt and intense glucose control.

EWDR has been reported in approximately 10%-20% of patients within 3-6 months after rapid improvement of blood glucose, with the main risk factor in individuals with pre-existing advanced baseline DR [14]. These changes were reported to be regressed after 12 months, with DR improvement by 18 months [15]. Other ocular diseases under speculation, similar to EWDR, are vitreous hemorrhage and NAION by outpacing the retina’s adaptive capacity. These adverse events are suggested to arise from acute vascular and structural stress on microvascular fragility and ultimately a rapid disruption in ocular homeostasis [16-19].

**Figure 2:**
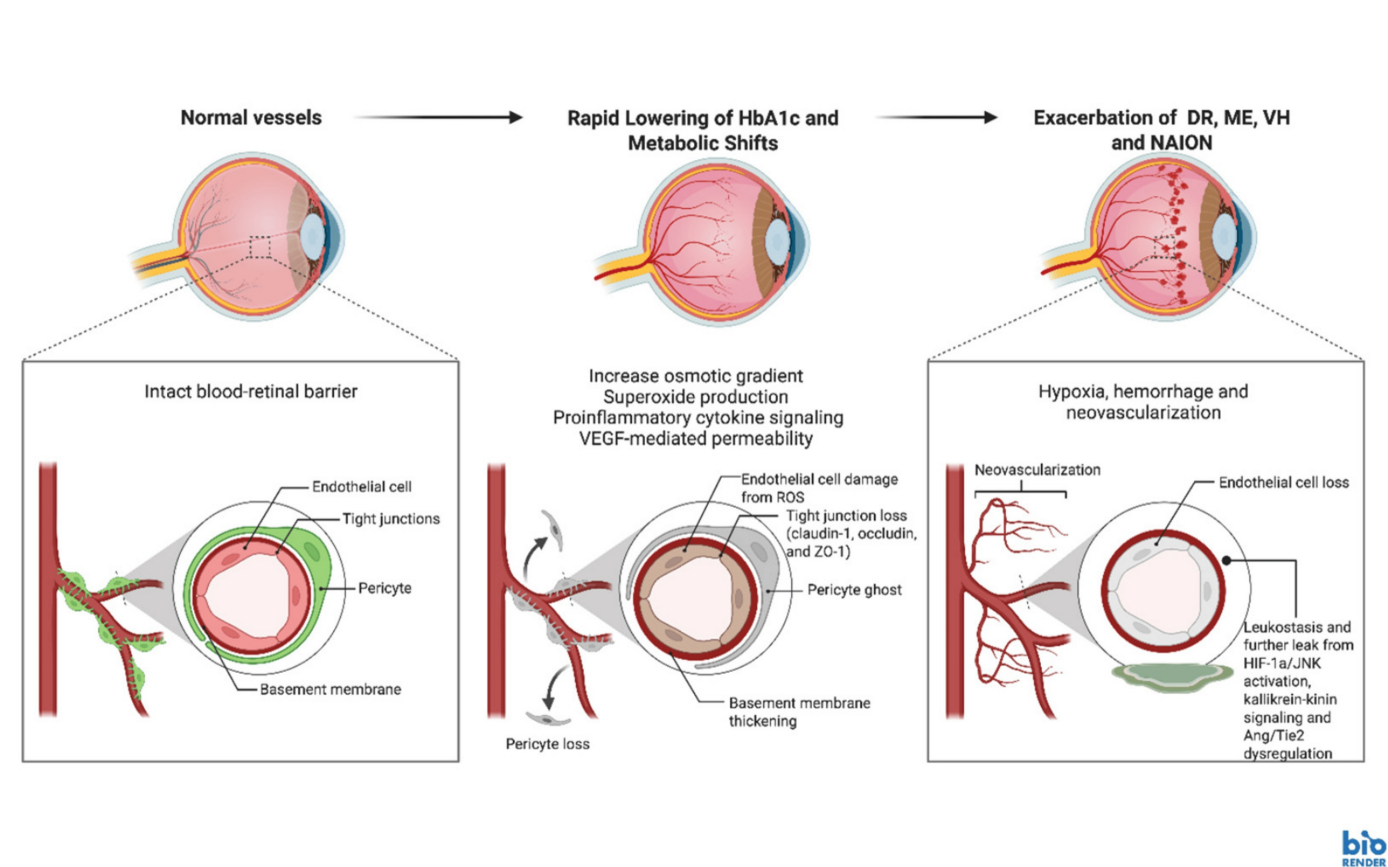
Vascular and metabolic effects of GLP-1RA on the ocular system Image Credit: Huang J. This image was created in BioRender (https://BioRender.com/zq0o7br). VEGF: vascular endothelial growth factor, ZO-1: zonula occludens-1, HIF-1a: hypoxia inducible factor 1-alpha, JNK: c-Jun N-terminal kinases, Ang/Tie2: angiopoietin/Tie2 receptor signaling pathway, DR: diabetic retinopathy, ME: macular edema, VH: vitreous hemorrhage, NAION: non-arteritic ischemic optic neuropathy, HbA1C: hemoglobin A1C, ROS: reactive oxygen species.

Under hyperglycemic conditions, glucose is rapidly converted into sorbitol through the polyol pathway. The accumulation of sorbitol leads to a disruption in the osmotic balance of cell membranes within the eye and ultimately leads to edema and cytotoxicity in ocular tissues [20]. Rapid glucose lowering with GLP-1RAs can lower intravascular osmotic pressure and create an osmotic gradient between the intravascular and extravascular space, which acutely stresses the retinal microvasculature. Ultimately, a larger and faster nonenzymatic glycated hemoglobin A1c (HbA1c) reduction and exposure to rapid acute lowering of vitreous glucose levels is hypothesized to induce endothelial dysfunction and mitochondrial oxidative stress. This pathological process has been associated with exacerbation of ischemic retinal injury and ocular edema [19,21]. Moreover, this metabolic shock amplifies endothelial oxidative stress, with brief lowering of blood glucose diminishing endothelial nitric oxide (NO) bioavailability and triggering mitochondrial superoxide production. These changes promote barrier instability and provoke systemic pro-inflammatory and prothrombotic responses, such as a rise in VEGF and adhesion molecules [22,23]. Hypoxia and cytokine signaling within the retina can drive up vascular endothelial growth factor (VEGF)-mediated permeability while remodeling tight-junction architecture (claudin-1, occludin, and zonula occludens-1) across the inner and outer blood-retinal barriers. The blood-retinal barrier (BRB) is a dual‑layer physiological barrier that protects the neural retina by tightly regulating molecular movement between the bloodstream and retinal tissue. It preserves retinal homeostasis, maintains immune privilege, and ensures optimal visual function. Loss of BRB integrity leads to retinal edema, neuroinflammation, and vision loss [24]. Hypoxia-inducible factor 1-alpha and c-Jun N-terminal kinase (HIF-1α/JNK) activation, kallikrein-kinin signaling, and angiopoietin/tyrosine kinase (Ang/Tie2) dysregulation further increase leak and leukostasis, leading to macular fluid accumulation [20,25]. This pathological mechanistic relationship is depicted in Figure 2.

This narrative review analyzed the current literature on GLP-1RAs and ocular diseases, emphasizing pathophysiology mechanisms from clinical findings and supportive evidence. By exploring the double nature of GLP-1RAs on the eye, this study aims to provide a foundation to understand the influence of GLP-1RAs on ocular disease onset and progression, and to better guide clinical decision-making and monitoring.

## Review

Methods

For this narrative review, PubMed, Elsevier, Embase, Google Scholar, Scopus, and Science Direct were accessed to retrieve relevant literature on September 13, 2024, and November 14, 2025, by two reviewers independently, and any disagreements were resolved by a third reviewer. The last search date was January 28, 2026. The search was limited to clinical trials, systematic reviews, meta-analyses, and observational studies published in peer-reviewed journals from 2014 to 2025. A Boolean search was conducted using a combination of the terms “ocular eye adverse effects events in GLP-1", “dulaglutide ophthalmic effects”, “semaglutide OR GLP-1 RAs”, and “glucagon-like-1 receptor agonist AND adverse effects”. For GLP-1RAs' effects on DR, the search terms used include “GLP-1 agonists AND diabetes”, "impact of GLP-1 AND diabetic retinopathy”, “diabetic retinopathy glycation”, “effects of GLP-1 on retina”, and "Retina AND GLP-1". For DME, the terms “the effects of GLP-1RA on macular edema” were used. For VH, the terms used were "GLP-1 AND vitreous hemorrhage”. For NAION, the terms utilized were "GLP-1RA and optic neuropathy”, “semaglutide optic neuropathy”, “GLP-1 AND NAION”, and “effects of GLP-1 on non-arteritic ischemic optic neuropathy”. For glaucoma and ocular hypertension, the terms used were "GLP-1RA AND glaucoma”. In RVO, the search included “GLP-1RA AND retinal vein occlusion” and “Effects of GLP-1RA on RVO”. And lastly, in idiopathic intracranial hypertension (IIH) and papilledema, the search terms used were “GLP-1 AND intracranial pressure”, “GLP-1 AND papilledema”, and “GLP-1 AND intracranial hypertension”. 

Inclusion Criteria

The search was limited to meta-analyses, clinical trials, systematic reviews, and case reports, reviews published in peer-reviewed journals relevant to GLP-1RAs' effects on ocular diseases. We extracted data on publication year, study design, authors, sample size, study outcomes, and key findings on the beneficial, neutral, and adverse effects of GLP-1RAs on specific ocular diseases. From selected studies, relevant information such as study population, proposed mechanisms, and outcomes was reviewed and summarized to provide a comprehensive overview of the effects of GLP-1RAs on the specific ocular disease.

Exclusion Criteria

Additionally, duplication studies, incomplete studies, and articles that were not in English were excluded. From there, a manual selection for appropriate journals related to GLP-1RAs on specific ocular diseases was done to ensure the inclusion of any additional publication or trials that might have been missed by the electronic search and the exclusion of any article that was ineligible or irrelevant to the narrative review. Ultimately, 60 studies set our criteria and were included in the review.

**Figure 3:**
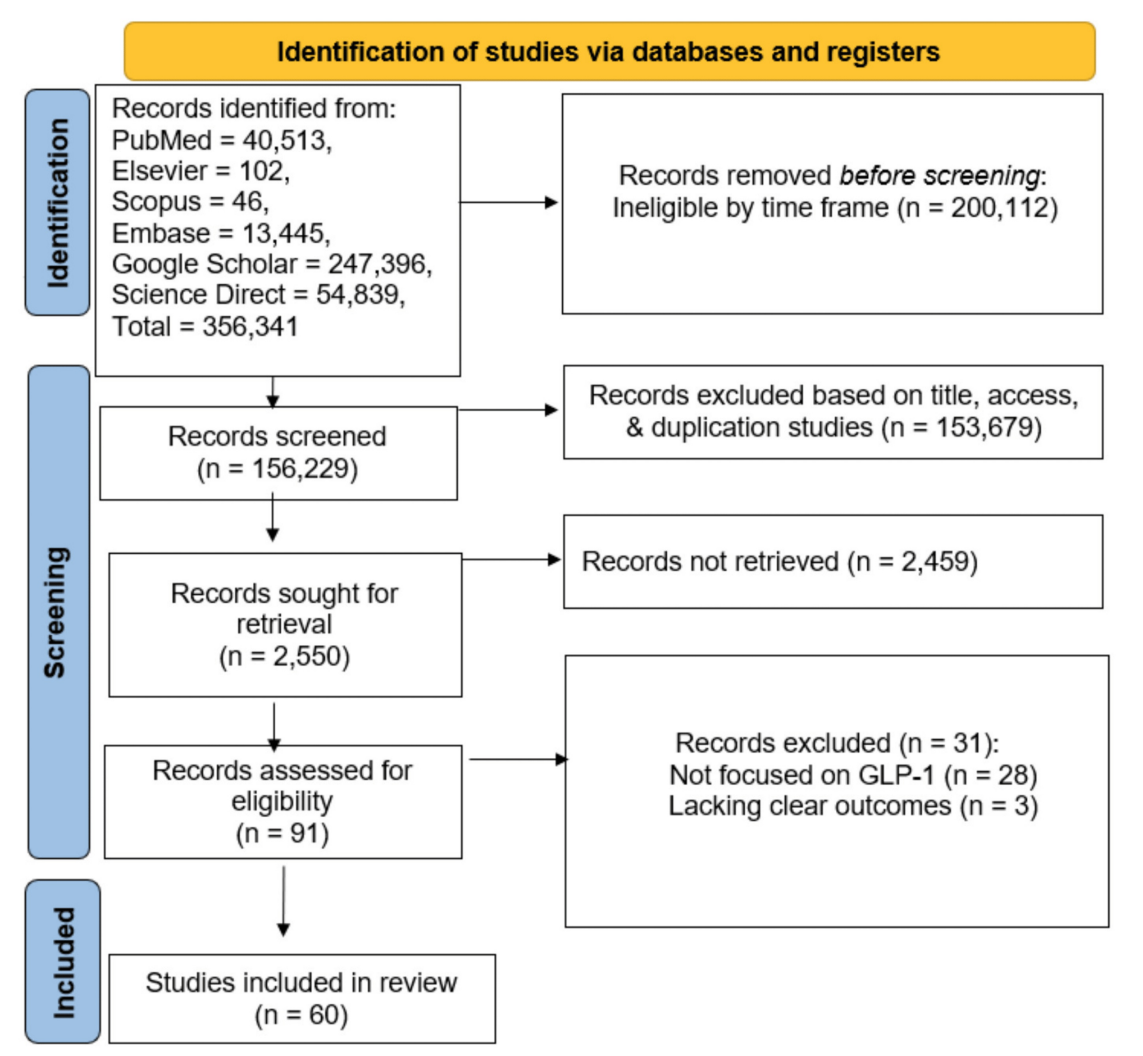
The search strategy and study selection process

The PRISMA guidelines were followed to minimize bias and maximize retrieval of pertinent studies [26]. The primary objective was to provide a comprehensive narrative synthesis of current evidence, rather than a quantitative aggregation or statistical evaluation. The study selection, inclusion, and exclusion process is presented in Figure 3.

Diabetic retinopathy

DR is a microangiopathy complication of diabetes mellitus affecting the retinal vasculature, resulting in potential visual impairment or blindness. DR is further classified into proliferative and non-proliferative DR. Classification criteria for proliferative retinopathy include extraretinal neovascularization with or without vitreous or preretinal hemorrhaging. Non-proliferative DR is subclassified based on the severity of microaneurysms, which differentiates between the severity of non-proliferative DR, with severe non-proliferative DR including venous beading and intraretinal microvascular abnormalities within the quadrants [27]. The pathogenesis underlying DR emphasizes damage occurring from hyperglycemia-mediated heavy oxidation from reactive oxygen species (ROS) to antioxidants. Hyperglycemia additionally leads to additional biochemical pathways that contribute to damage from ROS, including the polyol pathway, hexosamine pathway, protein kinase-C pathway, and accumulation of non-enzymatically produced advanced glycation end products (AGEs), ultimately leading to destruction of the blood-retinal barrier (BRB) and neo-vascularization [28].

GLP-1RAs are well studied for their metabolic benefits and glycemic modulation. The small number of studies with results on GLP-1RA use against DR outcomes, comparison studies of GLP-1RAs with other glucose-modulating medications such as sodium-glucose co-transporter 2 inhibitors (SGLT2i), and DR exacerbation associated with GLP-1RA usage present as novel research and prompt further investigation into current literature.

A meta-analysis on 93 trials of GLP-1RA and onset of early-stage DR analysis by Kapoor et al. demonstrated albiglutide to be responsible for the elevated risk of early-stage DR (RR = 2.18) versus placebos and demonstrated a decreased risk for late-stage DR (RR = 0.25, 95% CI 0.09, 0.70) when compared to insulin [16]. A retrospective study by Lin et al. (N = 97,413) compared GLP-1 RA to SGLT2i on DR progression and found a significantly higher incident rate of progression (sub-distribution hazard ratio (SHR): 1.50 (95% CI: 1.01-2.23) in the GLP-1RA group) [29]. Additionally, an aggregated electronic health record study (N = 6481) similarly compared monotherapy GLP-1RA to SGLT2i and noted a higher rate of DR progression during one year for the GLP-1RA monotherapy group (RR: 1.26) and three years of treatment (RR: 1.284, p = 0.002) [30]. Furthermore, a retrospective study using data from a global federated database (TriNetX, Cambridge, USA) compared the risk of DR in three separate groups of patients with T2DM: GLP-1 RAs + insulin (n = 207,034) treatment group, SGLT2i + insulin (n = 176,409) treatment group, and the insulin-only control group. The authors found a significantly increased risk of DR (1.308; 1.261, 1.357) compared to both the insulin-only group and the SGLT2i + insulin group (1.205; 1.153, 1.259) [31]. Although this study demonstrated GLP-1RA against insulin and SGLT2i, the effects of monotherapy GLP-1RA in relation to DR onset are unclear from this study. The absolute risk increase was small.

Notably, tirzepatide is a dual glucose-dependent insulinotropic polypeptide (GIP/GLP-1RA), and the increasing clinical use warrants specific discussion on whether the ocular findings related primarily to GLP-1RAs are associated with this subclass. A retrospective study using clinical data comparing type 2 diabetics exposed to tirzepatide to diabetic individuals not exposed to tirzepatide, with baseline considerations found a higher incidence of new-onset proliferative DR (PDR) in tirzepatide-exposed (1.1%, n = 33/3435) versus unexposed patients (0.5%, n = 17/3435) (OR 2.15; p < 0.01) [32]. The main limitation of this study is reduced applicability regarding GLP-1RA-naive participants starting tirzepatide and individuals whose baseline glycemic profile is not well-controlled. Although this study accounts for baseline glycemic profiles, the cohort consisted of individuals with relatively well-controlled glycemia. Thus, clinical decision-making should consider this limitation.

On the other hand, another study compared the rates of DR in SGLTi users (n = 21,491) to GLP-1RA users (n = 1887). The authors demonstrated comparable rates of any DR (SHR 0.90; 95% CI 0.79-1.03) and a significantly lower rate of proliferative DR (SHR 0.53; 95% CI 0.42-0.68) for the SGLTi group [33]. This study compared a select few SGLTi (dapagliflozin, canagliflozin, and empagliflozin) to GLP-1RAs (dulaglutide and liraglutide). A retrospective cohort study by Douros et al. sampled 77,115 patients with T2DM and did not show a significantly increased risk of DR incidence in the GLP-1RA cohort compared to two or more oral anti-diabetic medications (adjusted HR 1.00, 95% CI 0.85-1.17). Furthermore, comparison of data of GLP-1RA cohort against the insulin-only cohort demonstrated a lower risk of DR in the GLP-1RA cohort (adjusted HR 0.67, 95% CI 0.51-0.90). Notably, the authors highlighted a significant transient increase in DR risk during 6.1-12 months of GLP-1RA usage (HR 1.44, 95% CI 1.06-1.95), which eventually normalized after a year [34].

A possible explanation for the initial worsening of DR can be explained from two clinical trials: the LEADER trial (Liraglutide Effect and Action in Diabetes: Evaluation of Cardiovascular Outcome Results: A Long-Term Evaluation), involving liraglutide, and the SUSTAIN-6 trial (trial to evaluate cardiovascular and other long-term outcomes with semaglutide in subjects with T2DM), involving semaglutide [35]. The LEADER trial focused on patients with T2DM who were at elevated cardiovascular risk and demonstrated that liraglutide lowered the rate of cardiovascular morbidity and mortality. Notably, there was an increased but insignificant rate of DR events with liraglutide than with placebo. The SUSTAIN-6 trial similarly displayed beneficial cardiovascular outcomes of semaglutide. Notably, the trial found a significant increase in the rate of severe DR complications compared to placebo [36]. 

Overall, current meta-analysis and systemic reviews demonstrate inconclusive results regarding the relationship between GLP-1RAs and risk of DR. A study analyzing 23 randomized controlled trials (RCTs) evaluated the risk of DR with semaglutide treatment (n = 463) compared to the control group (n = 267) and reported that semaglutide was not associated with elevated DR risk when combining all 23 RCTs; however, when a subgroup analysis was performed, the authors’ analysis showed an elevated risk of DR in the semaglutide group versus the control [37]. A meta-analysis of 13 RCTs aimed to examine the effect of GLP-1RAs on DR with or without cardiovascular benefits in T2DM. They reported an elevated risk of worsening DR in 4 out of 13 RCTs with cardiovascular benefits with GLP-1RA (OR 1.23, 95 % CI 1.05-1.44). RCTs over a 52-week-long duration showed a significant association between GLP-1RAs and DR (1.2, 1.00-1.43) when compared to the placebo (1.22, 1.05-1.42) [7].

A common limitation among some of the studies above was the lack of detailed baseline glycemic profiling. In the meta-analysis studies, DR was evaluated as an adverse event rather than a primary outcome. Given a lack of baseline values, the prediction and correlation of clinical outcomes are difficult.

Current literature involving GLP-1RA treatment associated with DR and the meta-analysis of the literature reflect conflicting results, justifying further research to potentially achieve conclusive results. The observed literature in support of DR worsening highlights the necessity to consider the potential adverse effects of medication choice. The findings are summarized in Table 1.

**Table 1:** Effects of GLP-1RAs on diabetic retinopathy DR: diabetic retinopathy, CVOT: cardiovascular outcome trial, EHR: electronic health record, RCTs: randomized controlled trials, SGLT-2i: sodium-glucose linked transporter inhibitor; GLP-1 RA: glucagon-like peptide-1 receptor agonist.

Author (year), country	Study population	Benefits (B)/neutral (N)/risk (R)
Yoshida et al. (2022) [7], United States	N = 2519	R: RCTs showed an elevated risk of DR with GLP-1 RAs only in CVOT trials. RCTs longer than 52 weeks and RCTs comparing GLP-1RA to placebo showed an increased risk of DR with GLP-1 RA
Kapoor et al. (2023) [16], multinational	N= 106,819	R: Albiglutide showed increased risk of early-stage DR in GLP-1 RA compared to the control and lower risk for late-stage DR in GLP-1 RA compared to insulin
Lin et al. (2024) [29], Taiwan	N = 97,413	R: Increased DR progression in GLP-1 RA compared to SGLTis. Baseline DR severity was not fully controlled
Wai et al. (2024) [30], United States	N = 6481	R: Higher rate of DR progression in monotherapy GLP-1 RA against SGLT2i for 1 and 3 years. The study used EHR coding, which may under-capture DR severity
Eleftheriadou et al. (2024) [31], global	N = 2,305,755	R: GLP1-RA + insulin cohort increased risk for DR compared to insulin only. The GLP1-RA + insulin cohort increased risk for DR compared to the SGLTis + insulin cohort
Buckley et al. (2025) [32], United Kingdom	N = 3435	R: Increased risk of new-onset proliferative DR occurred in tirzepatide-exposed individuals compared to non-exposed individuals when adjusted for established risk factors
Lin et al. (2023) [33], Taiwan	N = 23,378	R: Lower rate of progressive DR in the SGLTi group compared to GLP-1 RA
Douros et al. (2018) [34], United Kingdom	N = 77,115	N: No increased risk in GLP-1 RA compared to ≥2 antidiabetic medications. GLP-1 RA had a lower risk of DR compared to insulin. At 6.1-12 months, GLP-1 RA transiently increased DR risk. Beyond 12 months, the risk normalized. The absolute risk remained low
Simo et al. (2017) [36], Spain	N = 12,637	N: DR events in liraglutide were not significantly higher than placebo. Semaglutide significantly increased the rate of DR compared to placebo. Early transient worsening of DR in type 1 diabetics
Wang et al. (2022) [37], China	N = 22,096	N: No associative increase in risk of DR with GLP-1 RA compared to the control when all trials were combined. Subgroup analysis showed increased risk with semaglutide

Diabetic macular edema

DME is the accumulation of fluid, extrapolated from leaky blood vessels, in the macula of a diabetic patient. This pathological process leads to a thickening of the retina and is a significant cause of vision impairment in diabetic patients. Although DME is almost always associated with or considered a complication of DR, its pathogenesis is not entirely understood. The understanding of DME is in two forms: cytotoxic and vasogenic. Cytotoxic DME results from increased sorbitol, lactate, and phosphate in the intracellular space secondary to hyperglycemia [38]. The vasogenic form begins with hypoxia in the retina, causing capillaries to become hyperpermeable. The increase in permeability is due to a rise in vascular endothelial growth factor (VEGF) in response to hypoxia. As such, current treatments include constant injections of anti-VEGF agents. However, patients may not respond well to these injections, which can lead to a high prevalence of DME. This also suggests that unknown factors other than VEGF are associated with the condition. Therefore, the use of GLP-1RAs has been investigated for the direct treatment of DME and DME-associated DR [1]. In evaluating the relationship between glucose-lowering agents and DME, an EMR study compared the effects of GLP-1RAs on SGLT-2i on DR and DME. After propensity score matching, a total of 6481 patients were identified in the GLP-1RA cohort and the SGLT-2i cohort. Patients who received monotherapy with GLP-1RA showed an increased risk of developing new-onset DME compared to those treated with SGLT-2i. The GLP-1RA group demonstrated an increased rate of DME at three months (RR 1.192, p = 0.002), six months (RR 1.22, p < 0.001), one year (RR 1.24, p < 0.001), and three years (RR 1.29, p < 0.001) [30].

Rapid and significant reduction in HbA1c is speculated to cause sudden worsening of DR and be associated with macular edema. In a case study by Brooks et al., a 36-year-old woman with T2DM experienced significant HbA1c reduction from 11.9% to 4.8% over just four months following exenatide therapy, alongside considerable weight loss [39]. Despite these metabolic improvements, she progressed from background retinopathy to bilateral proliferative DR with DME requiring urgent laser treatment. The paradoxical nature of pathophysiology is not fully understood; however, the author suggested the cause to be a reduction in retinal blood flow and an increase in insulin-like growth factors (IGF-1), followed by rapid glycemic control, which may trigger retinal ischemia or growth factor-mediated vascular changes that exacerbate DME. The authors hypothesized that GLP‑1RAs may aggravate existing retinal microvascular disease, potentially via neuroinflammatory pathways, although these mechanisms were not directly measured [39].

A recent retrospective cohort study using real-world data further explores the association between DME and glucose-lowering therapy by comparing GLP-1RAs and SGLT-2i in patients with DR and DME. The study found that compared with SGLT-2i plus insulin, GLP-1 receptor agonists plus insulin were associated with an elevated risk of DR and DME (1.130; 95% CI 1.056, 1.208; χ^2 ^= 0.006). The analysis of the study used a propensity score that matches certain variables such as age, sex, comorbidities, HbA1c, BMI, and medications. The findings of the study suggest that GLP-1RAs are hypothesized to contribute to retinal neuroinflammation and aggravate existing retinal microvasculature disease [31].

A slower titration protocol can be considered in patients with advanced DR/DME. Studies utilizing a slower titration showcase fewer adverse gastrointestinal effects and uncompromised efficacy of semaglutide in patients with T2DM. Specifically, instead of increasing the dosage from 0.25 mg, 0.5 mg, and 1 mg over four-week intervals, patients were started at 0.0675 mg with an increase of 0.0675 mg every week for 26 weeks [40]. This regimen provides circumstantial evidence that a similar, decreased rate of GLP-1RA administration in relation to eye pathology may provide beneficial effects. These findings are summarized in Table 2.

**Table 2:** Effects of GLP-1RAs on diabetic macular edema GLP-1RA: glucagon-like peptide-1 receptor agonist, SGLT-2i: sodium-glucose linked transporter-2 inhibitor, DME: diabetic macular edema, HbA1c: hemoglobin A1c.

Author (year), country	Study population	Benefits (B)/neutral (N)/risk (R)
Wai et al. (2024) [30], United States	N = 6481	R: An increased rate of progression of PDR and increased risk of DME with GLP-1 agonists vs SGLT-2i
Eleftheriadou et al. (2024) [31], multinational	N = 2,305,755	R: GLP-1RA is associated with an elevated risk of DME when compared to SGLT-2i

Vitreous hemorrhage

Vitreous hemorrhage (VH) is associated with a multitude of etiologies, such as PDR, ocular trauma, vascular occlusion, retinal breaks, and retinal detachment. In a pathophysiological context, VH is associated with rupture in abnormal ocular vessels and extravasation of blood into the vitreous cavity and adjacent structures. As discussed in the effects of GLP-1RAs in DME, rapid glycemic correction associated with GLP-1RA therapy may disrupt vascular integrity. With emerging clinical observation, the potential of developing or exacerbating VH with the use of GLP-1RAs warrants further investigation.

A retrospective case-control study analyzed 35 GLP-1RA users against 31 control patients, demonstrating a higher prevalence of VH in GLP-1RA users compared to controls (p = 0.023), but the sample size was very small [41]. A single-patient case study reports a case of VH with pre-existing DR during reinitialization and up-titration of dulaglutide. The study highlights the rapid overcorrection of HbA1c reduction by 1.5%. The authors report that the patient has stable hypertension management with no posterior vitreous detachment or ocular trauma that would suggest the cause of VH. With a Naranjo score of 2, these findings explain the possibility of VH related to dulaglutide use. However, causality cannot be established as the authors also highlighted the possibility of VH development from worsening DR rather than GLP-1RA use [42]. A study on 60 RCTs with 60,077 patients to assess the microvascular effects of GLP-1 RAs users. The incidence of VH was found to be significantly elevated in patients treated with GLP-1 RAs versus placebo, citing an association primarily driven by the 2016 LEADER trial, which investigated the association between liraglutide and cardiovascular outcomes of patients with T2DM [42-44]. In contrast, a retrospective cohort study of 1191 patients who have either taken GLP-1RA (n = 692) or SGLT-2i (n = 289) between 2012 and 2023 showed that most of the clinical worsening cases were patients who subsequently developed VH on GLP-1RA (n = 7, 43.8% of worsening events) However, the odds of VH or retinal detachments were not statistically different between GLP-1RA users and controls [45]. Given that VH most commonly arises from PDR, the observed association in these studies may be reflecting disease progression or destabilization of underlying PDR, an indirect effect of transient rapid glycemic improvement rather than a direct drug-specific effect of GLP-1RAs.

Despite these emerging studies, the current body of evidence evaluating VH in the context of GLP-1RA use remains limited and mixed. The variability in study design and confounding baseline ocular pathologies, such as DR severity, limits causality. The recurring clinical cases of VH in several GLP-1RA studies highlight the importance of close monitoring, especially in patients with pre-existing retinal disease. These findings are summarized in Table 3.

**Table 3:** Effects of GLP-1RAs on vitreous hemorrhage GLP-1RA: glucagon-like peptide-1 receptor agonist, VH: vitreous hemorrhage.

Author (year), country	Study population	Benefits (B)/neutral (N)/risk (R)
Michaeli et al. (2024) [41], Israel	N = 66	R: Higher prevalence of vitreous hemorrhage in GLP-1RA users vs controls
Avgerinos et al. (2019) [43], multinational	N = 60,077	R: Increased odds of vitreous hemorrhage
Joo et al. (2024) [45], South Korea	N = 1191	N: VH was the most common clinical worsening event in GLP-1RA users (43.8%); odds of VH were not statistically significant

Non-arteritic anterior ischemic optic neuropathy

NAION is currently one of the causes of acute optic nerve injury in individuals past the age of 50. This sudden, painless loss of monocular vision is due to an obstructed blood supply to the anterior part of the optic nerve. Although the pathogenesis and treatment plans are not well studied, the pathophysiology of NAION is hypothesized to be a reduction of blood flow in the short posterior ciliary arteries by the optic nerve head, which may contribute to optic nerve swelling. As a result, the swelling further constricts microcirculation and ultimately leads to apoptosis of the retinal ganglion cells. While there are a growing number of reports possibly linking GLP-1RA to the risk of NAION, it is necessary to acknowledge that patients receiving GLP-1RA medication overlap substantially with those already at a baseline risk for NAION due to pre-existing medical conditions, including diabetes and hyperlipidemia. As such, current literature indicates GLP-1RAs as an emerging risk signal for NAION, and future prospective studies investigating a potential association must carefully consider confounding variables. Still, as NAION can lead to irreversible vision loss and the use of GLP-1RAs has rapidly increased over the past decade, further investigation is warranted [46].

One of the first studies to introduce the potential association between GLP-1RAs and NAION is a retrospective matched cohort study. It calculated an HR of 4.28 (95% CI 1.62-11.29, p < 0.001) in semaglutide users with T2DM compared to the non-semaglutide cohort over 36 months. When comparing the incidence of NAION in semaglutide users who were overweight or obese within the same timeframe, Hathaway et al. also showed a cumulative incidence of NAION of 6.7% for the semaglutide cohort, compared to 0.8% in the control. As a result, the COX proportional hazards regression model showed a higher risk of NAION for patients in the semaglutide cohort (HR of 7.64, p < 0.001), and event numbers were extremely small [6].

The risk signals observed by Hathaway et al. [6] raise concerns related to a potential association between the use of semaglutide and the onset of NAION. However, causality cannot be concluded as the included semaglutide users share established risk factors for NAION (e.g., diabetes, hypertension, and obstructive sleep apnea). Despite the use of propensity matching, unmeasured variables (e.g., duration of diabetes) present the possibility of substantial residual confounding, commonly seen in retrospective studies.

Following their findings that semaglutide users’ risk for developing NAION was roughly 4-7 times more than that of non-semaglutide users, a five-year longitudinal cohort study by Grauslund et al. also found that once-weekly use of semaglutide more than doubled the risk of NAION [17]. Moreover, the study by Grauslund et al. adjusted for various confounders, including duration of diabetes, hemoglobin A1c, estimated glomerular filtration rate, cardiovascular disease, insulin, use of cholesterol medicine, and use of antihypertensives during the sensitivity analyses. During 294,395 person-years of observation, there were 67 incidences of NAION with GLP-1RA exposure (HR 2.57, 95% CI 1.92-3.45). This figure was 2.19 times higher than the non-exposed cohort, which had 151 incidences across 1,620,725 person-years (incidence rate of exposed 0.228 versus not-exposed 0.093 per one thousand person-years, p < 0.001), and NAION incidence remained low [17].

Another study found consistent results that demonstrated a nearly threefold increase in NAION risk with the adjusted pooled HR of 2.81 (p < 0.05) and an IRD of +1.41/10000 person-years (95% CI +0.53 to +2.29) for semaglutide users in comparison to SGLT-2i users. Confounding was reduced through propensity scores that adjusted for markers of diabetes severity and systemic risk factors for NAION, including obesity and smoking. A post hoc analysis also adjusted for duration of diabetes and yielded near-exact results, suggesting that diabetes severity may have a limited impact on the results of the study. Although this finding supported an association between semaglutide use and an elevated risk of NAION, Simonsen et al. also noted that the overall absolute risk of NAION remains low with semaglutide users [47].

Most recently, the largest study on this potential relationship, across 14 databases and 37.1 million patients, also found an increased risk of NAION in semaglutide users, a slight increase in risk in exenatide users, and no increase in risk for non-GLP-1RA users [48]. Specifically, for semaglutide users, a meta-analysis revealed an IRR of 1.32 (95% CI, 1.14-1,54, p < 0.001) [48]. Confounding factors were adjusted through the use of negative control outcomes.

All four retrospective studies encountered similar difficulties with their electronic health record (EHR) databases. Each study cited difficulties in finding detailed clinical data that limited both the capture of confounders and the number of true NAION events due to EHR coding misclassification or inconsistency. As EHR-related issues are a common limitation in these four retrospective studies, causality cannot be established.

Finally, several case reports have documented similar cases of NAION onset with semaglutide use. In a 2024 case report by Karam et al., a 73-year-old man with a history of T2DM, metabolic syndrome, and cardiac illness was recently prescribed semaglutide [49]. The first day after increasing the dosage from 1 mg to 2 mg, the patient experienced painless visual loss in the left eye, and an ophthalmologist subsequently confirmed a diagnosis of NAION. Three weeks later, the patient experienced a similar event in the right eye. As current studies point toward poor vascular perfusion of the optic disc as a cause for NAION, the authors suspect that the patient’s significant weight loss and postural hypotension that accompanied semaglutide use may have contributed to the bilateral NAION onset [49].

Another 2025 report by Mao et al. also presented a distinct case of a 63-year-old man with a history of essential hypertension, hyperlipidemia, T2DM, hypothyroidism, and sleep apnea, who was diagnosed with NAION OD and developed permanent vision loss after increasing his semaglutide dosage from a weekly 0.25 mg to 0.5 mg [50]. While on semaglutide, the patient’s HbA1c rapidly reduced by 1.3% over four months, but this was excluded as a potential cause for NAION, as the patient had no pre-existing history of diabetic retinopathy. Additionally, nocturnal hypoperfusion due to antihypertensive medications was not likely, as the patient was also taking nightly metoprolol succinate, a beta-blocker that does not peak within 4-6 hours of sleep. After ruling out other possible etiologies, semaglutide use was considered a possible contributing factor to the onset of the patient’s NAION [50].

Establishing a causal relationship between the onset of the disease and the use of GLP-1RA is challenging since the pathophysiology of NAION is still unclear, and there is substantial overlap surrounding GLP-1RA users and the major risk factors for NAION, complicating future studies investigating any potential linkage between the two. There is currently a lack of ongoing prospective studies and a lack of uniform data that limits researchers from exploring concrete conclusions. However, the increase in recent reports indicates that GLP-1RAs must be considered an emerging risk signal for NAION. Most recently, in June 2025, the European Medicines Agency (EMA) concluded, based on all available data from non-clinical studies, clinical trials, literature, and post-market surveillance, that NAION is a very rare side effect of semaglutide. As such, EMA subsequently recommended that the product information for all semaglutide medicines be updated to reflect this decision [51]. Regardless, providers must recognize early signs of vision loss in GLP-1RA users or users who have recently increased their dosage.

Lastly, it is important to note that NAION is considered a relatively rare condition, affecting roughly 2-10 per 100,000 people in the United States annually. While the statistical findings from this report present hazard ratios suggesting a relative increase in NAION risk among semaglutide users, the absolute risk increase remains low among semaglutide users. As such, the long-established benefits of semaglutide should be considered in the treatment of diabetes and obesity, even when investigating a possible association with NAION [52]. These findings are summarized in Table 4.

**Table 4:** Effects of GLP-1RA on non-arteritic anterior ischemic optic neuropathy NAION: non-arteritic anterior ischemic optic neuropathy, GLP-1RA: glucagon-like peptide-1 receptor agonist.

Author (year), country	Study population	Benefits (B)/neutral (N)/risk (R)
Hathaway et al. (2024) [6], multinational	N = 1903	R: Higher risk of NAION in semaglutide users
Grauslund et al. (2024) [17], Denmark	N= 424,152	R: Low incidence rate of NAION in GLP-1RA users
Simonsen et al. (2025) [47], Denmark and Norway	N = 44,517	R: Although relative risks for NAION appear increased with semaglutide in several cohorts, the absolute incidence remains low; causality has not been definitively established
Cai et al. (2025) [48], multinational	N = 37.1 million	R: Meta-analysis reveals an increased risk of NAION due to semaglutide exposure
Karam et al. (2024) [49], USA	N = 1	R: NAION OU new onset after increasing semaglutide dosage from 0.25 mg per week to 2 mg per week. The patient’s weight loss with semaglutide and resulting postural hypotension may have contributed to NAION onset
Mao et al. (2025) [50], USA	N = 1	R: NAION OD new onset after increasing semaglutide dosage from 0.25 mg to 0.5 mg. Other potential causes, including rapid A1C reduction and nocturnal hypoperfusion, were ruled out

Glaucoma and ocular hypertension

Glaucoma is a disease of the eyes that can cause optic nerve damage, loss of retinal cells, and an enlargement of the optic cup. While this condition is commonly linked to increased intraocular pressure (IOP), glaucoma can also occur when IOP levels are normal.

Risk factors include age, elevated IOP, family history, ethnicity, and myopia. The two most common types of glaucoma are acute angle closure and open-angle glaucoma. In acute angle closure, the Canal of Schlemm, which drains aqueous humor, becomes blocked due to the iris pressing against the trabecular meshwork. This type of glaucoma tends to present symptoms including pain, loss of peripheral vision, a hard globe, and hyperemia. In contrast, open-angle glaucoma is characterized by an elevated IOP without any blockage, with symptoms emerging only in the advanced stages of the disease. Treatments for glaucoma include miotic agents, beta blockers, alpha-2 agonists, carbonic anhydrase inhibitors, mannitol, prostaglandin analogs, laser trabeculoplasty, cyclophotocoagulation, cyclocryocoagulation, insertion of stents, sclerotomy, and trabeculectomy [53].

GLP-1RAs have been associated with positive findings for glaucoma. In a retrospective cohort study conducted on GLP-1RAs, users were compared to a control group of 4371 people. There were 10 new cases of glaucoma in the GLP-1RA group and 58 new cases in the control group. Ultimately, the researchers obtained a hazard ratio of 0.58 (p = 0.01), showcasing a significant hazard reduction for glaucoma when using GLP-1RAs [54].

Further supporting the potential ocular benefits of GLP-1RAs, a recent large-scale cohort analysis showed that these agents significantly reduce the risk of ocular hypertension (OHT), primary open-angle glaucoma (POAG), and the initiation of first-line glaucoma treatments in patients with T2DM. Compared to metformin, patients treated with GLP-1RAs exhibited a 41% lower risk of developing POAG within the first year of treatment, which deepened to a 50% risk reduction by the second year and remained consistent at three years (RR = 0.59, 0.50, and 0.59, respectively). Similarly, the incidence of OHT was substantially reduced at all measured timepoints, with relative risk reductions of 56%, 57%, and 49% at one, two, and three years, respectively [11]. Notably, the need for early glaucoma interventions such as topical IOP-lowering medications or laser trabeculoplasty was also significantly lower in the GLP-1RA group, with a 37% decrease observed at one year, and continued benefit at two and three years.

A meta-analysis by Asif et al. describes findings related to how GLP-1RAs affect the incidence of glaucoma and how they compare to other glycemic control agents when treating glaucoma. The authors analyzed five well-researched studies that totaled 2,500,430 participants. Through their findings, GLP-1RAs reduced the incidence of glaucoma, albeit a nonsignificant reduction. These outcomes emphasize GLP-1RAs' impact as a preventative agent for glaucoma, rather than focusing on their treatment potential. Additionally, the authors concluded that GLP-1RAs are potentially more beneficial in treating glaucoma when evaluated alongside other diabetic medications (p = 0.01) [55].

The findings of these studies indicate that GLP-1RAs, in addition to their beneficial metabolic effects, provide a neuroprotective and anti-inflammatory benefit for the optic nerve and retinal ganglion cells [50]. These results highlight the observational benefits of GLP-1RAs in glaucoma treatment. These findings are summarized in Table 5.

**Table 5:** Role of GLP-1RAs in the management of glaucoma GLP-1RA: glucagon-like peptide-1 receptor agonist; POAG: primary open-angle glaucoma.

Author, Year, Country	Study Population	Benefits (B)/Neutral (N)/Risk (R)
Muayad et al. (2025) [11], United States	N = 61,998	B: GLP-1RAs significantly reduced the risk of developing POAG
Sterling et al. (2023) [54] United States	N = 6,332	B: The GLP-1RA group showed significant hazard reduction for glaucoma
Asif et al. (2025) [55], Pakistan and Sudan	N = 2,500,430	N: GLP-1RAs showed a nonsignificant reduction in the incidence of glaucoma

Retinal vein occlusion

Retinal vein occlusion (RVO) is the second most prevalent disorder after DR, comprising central (CRVO) and branch (BRVO) subtypes. GLP-1RAs have been postulated to restore glucose homeostasis in T2DM patients and reduce the incidence of small vessel occlusions by decreasing oxidative stress and microvasculature inflammation. The direct effects of how GLP-1RAs affect RVO have yet to be elucidated, and these mechanistic pathways are speculative. Evaluating antidiabetic drug classes in relation to RVO is clinically important given that glucose-lowering therapies have varying data on microvascular outcomes. A retrospective cohort study by Pan et al. (N = 79,486) revealed GLP-1RA use is associated with a decreased risk of RVO (HR 0.73; 95% CI 0.54-0.98) and BRVO (HR 0.62; 95% CI 0.41-0.95) over five years compared with DPP-4 inhibitor use. The authors concluded that using GLP-1RA in high-risk T2DM populations is beneficial over DPP-4 inhibitors [8]. In contrast, a nationwide propensity score study by Lee et al. showed that SGLT2i initiation was associated with a higher RVO risk (HR 1.264, 95% CI 1.056-1.513) [56]. This study prompts further evaluation of the effects of GLP-1RAs on RVO. Currently, there are no studies to delineate the mechanistic pathway of how GLP-1RAs affect RVO. Given these divergent studies across distinct antidiabetic classes, there is an unmet need for additional research to confirm and extend these findings. These findings are summarized in Table 6.

**Table 6:** Effects of GLP-1RAs vs DPP-4i in retinal vein occlusion GLP-1RA: glucagon-like peptide-1 receptor agonist, RVO: retinal vein occlusion, DPP-4i: dipeptidyl peptidase-4 inhibitor.

Author (year), country	Study population	Benefits (B)/neutral (N)/risk (R)
Pan et al. (2025) [8], multinational	N = 79,468	B: Lower RVO risk with GLP-1RA users compared to DPP-4i users

Idiopathic intracranial hypertension and papilledema

IIH is a neurological disorder characterized by elevated intracranial pressure (ICP) and is highly associated with papilledema and visual disturbances that may lead to permanent vision loss. Weight gain is the major factor associated with this condition. Mechanistically, GLP-1RAs appear to act through both systemic metabolic effects and direct modulation of cerebrospinal fluid (CSF) dynamics. In IIH, GLP-1RAs are effective for achieving weight loss, a factor that is significantly correlated with inducing disease remission. GLP-1RAs have been investigated as a potential adjunctive therapeutic strategy for IIH [57].

A randomized placebo-controlled double-blind trial by Mitchell et al. of adult women (N = 15) with active IIH (ICP > 25 cm CSF with papilledema) demonstrate subcutaneous exenatide significantly lowered telemetrically monitored ICP at 2.5 h (-5.7 ± 2.9 cm CSF; p = 0.048) and 24 h (-6.4 ± 2.9 cm CSF; p = 0.030), with a similar reduction at 12 weeks (p = 0.058) [58].

A retrospective cohort analysis compared 193 patients with IIH who were exposed to tirzepatide with 193 controls receiving standard care. They analyzed the severity of papilledema, visual function, headache frequency, and treatment resistance at multiple time points. Their analysis showed significant improvement in all measured outcomes in the tirzepatide group, with 68% reduction in papilledema risk at 24 months (p < 0.001) and a 73.9% reduction in visual disturbance and blindness risk (p < 0.001). Their study demonstrated that tirezepatide (GLP-1RA), an adjunctive therapy, significantly benefits IIH and papilledema management [59].

Even though dual agonists share beneficial effects of lowering ICP and papilledema risk, the dual agonist can produce larger and more rapid HbA1c reductions than GLP-1RAs. This effect carries an “EWDR” risk profile as discussed in the DR section [32] 

In another retrospective cohort study, 204 patients with IIH receiving liraglutide were compared to 204 patients with standard IIHC therapy, demonstrating a significant reduction in papilledema risk at three months (p = 0.001), with sustained benefits throughout two years (p < 0.05). This study supports and strengthens GLP-1RAs as a beneficial adjunctive therapy for improving IIH, particularly papilledema [23]. More recently, an extensive retrospective cohort study (N = 44,373 patients identified; after matching, n = 555 GLP-1RA users vs n = 555 nonusers with one-year follow-up) found that GLP-1RA users have reduced papilledema (p < 0.01) [60]. Collectively, these studies spanning from small RCTs to multiple real-world cohort observations indicate a beneficial association between GLP-1RA therapy and papilledema, ultimately supporting further prospective evaluation and adjunctive therapy consideration in IIH. These findings are summarized in Table 7. Overall benefits and risks of GLP-1Ras on ocular diseases are summarized in Table 8 and Table 9.

**Table 7:** Effects of GLP-1RAs on idiopathic intracranial hypertension and papilledema GLP-1RA: glucagon-like peptide-1 receptor agonist, ICP: intracranial pressure, IIH: idiopathic intracranial hypertension.

Author (year), country	Study population	Benefits (B)/neutral (N)/risk (R)
Azzam et al. (2025) [23], multinational	N = 408	B: Liraglutide adjunctive treatment in IIH patients showed papilledema risk reduction for three months
Mitchell et al. (2023) [58] United Kingdom	N = 15	B: Exenatide lowered ICP at 2.5 h and 12 weeks with no serious safety signals
Azzam et al. (2024) [59], multinational	N = 383	B: Tirzepatide adjunctive therapy in IIH patients showed improvements in papilledema, visual disturbance/blindness, and headache relative risk at 24 months
Sioutas et al. (2025) [60], United States	N = 1110	B: Decreased medication use, headaches, visual problems, hospital visits, and lower mortality in GLP-1RA uses

**Table 8:** Overall benefits and risks of GLP-1RAs on the ocular system VEGF: vascular endothelial growth factor, NAION: non-arteritic anterior ischemic optic neuropathy.

Benefits	Risks
Improve retinal vascular regulation and reduce permeability	Rapid glycemic correction leads to transient worsening of diabetic retinopathy (DR)
Anti-inflammatory effects: preserve the blood-retinal barrier, reduce VEGF signaling	Osmotic stress from sorbitol accumulation leads to edema and cytotoxicity
Neuroprotective: attenuates microglial activation, supports retinal ganglion cell survival	Oxidative stress and endothelial dysfunction lead to VEGF upregulation and barrier instability
Lower intraocular and intracranial pressure leads to potential benefits in glaucoma and idiopathic intracranial hypertension (IIH)	Microvascular fragility leads to vitreous hemorrhage, NAION

**Table 9:** Overall effect of GLP-1RA on ocular diseases RCT: randomized controlled trial, LEADER: Liraglutide Effect and Action in Diabetes: Evaluation of Cardiovascular Outcome Results, SUSTAIN-6: The preapproval Trial to Evaluate Cardiovascular and Other Long-term Outcomes with Semaglutide in Subjects with Type 2 Diabetes, GLP-1RAs: glucagon-like peptide-1 receptor agonists, DPP-4i: dipeptidyl peptidase inhibitor, SGLT-2is: sodium glucose co-transporter 2 inhibitors, ICP: intracranial pressure, POAG: primary open-angle glaucoma.

	Ocular disease	Reference	Findings
1	Diabetic retinopathy (DR)	Meta-analyses and RCTs (LEADER; SUSTAIN-6; [7], [16], [35], [44])	Mixed: transient worsening with semaglutide/liraglutide; some studies show increased progression vs SGLT2i
2	Diabetic macular edema (DME)	Case reports [30,31]	Higher risk of new-onset DME with GLP-1RA, especially with rapid HbA1c reduction
3	Vitreous hemorrhage (VH)	Case reports [41,43]	Association with VH, though causality unclear; risk is higher in pre-existing DR
4	NAION	Case reports [17,49,50]	Emerging risk linked with semaglutide; dose escalation implicated
5	Glaucoma/OHT	[10,11,54,55]	Protective: reduced incidence of POAG and ocular hypertension
6	Retinal vein occlusion (RVO)	[8,56]	Current data do not suggest increased RVO risk with GLP‑1RAs; one large cohort shows lower risk vs DPP‑4i
7	Idiopathic intracranial hypertension (IIH)	Exenatide RCT [58]; [23,59]	Reduced ICP and papilledema; promising therapeutic role

Limitations

This narrative review has several limitations in both design and the heterogeneity of the available studies. The literature evaluating DR outcomes with GLP-1RAs is conflicting, with studies reporting either transient effects on disease progression or no association. A formal meta-analytic approach was not performed due to the heterogeneity across studies, such as differences in study design, control groups, defined outcomes, duration of follow-up, and whether DR was assessed as a primary endpoint or reported as only an adverse event. These studies differ in the information provided, such as standardized, consistent baseline retinopathy grading and consistent reporting of dose escalation and titration timing. A structured qualitative synthesis is used to emphasize the potential risks given by these constraints. Another limitation is the predominance of retrospective studies. These studies rely on electronic health records and ICD-based diagnoses, which may obscure substantial confounding potential that is related to diabetes duration and disease severity, such as hypertension, vascular disease, and sleep apnea. Lastly, there is limited data on the effects of GLP-1RAs on VH and the ocular relationship of newer agents such as GIP/GLP-1 dual agonists. These circumstances restrict definitive risk estimation and emphasize the need for future studies that have standardized endpoints.

## Conclusions

This review highlights a complex and bidirectional relationship between GLP-1RAs and ocular diseases. The benefits of GLP-1RAs include decreased risk of ocular hypertension and POAG by decreasing neuroinflammation and promoting retinal ganglion cells rescue by glial activation. There is also encouraging data on the therapeutic advantages and neuroprotective effects of GLP-1RAs in IIH and papilledema by weight loss, reducing CSF production, and ultimately inducing disease remission. Across the ocular conditions reviewed, GLP-1RAs present with observational associations of early and transient worsening of DR, alongside downstream risks of DME, VH, and NAION. Consider slower titration in patients with advanced DR/DME and screen for baseline ocular disease before initiating GLP-1RA. 

Mechanistically, these different outcomes may be explained by the harmful effects of rapid glycemic shifts, causing osmotic stress and ultimately a destabilization of the retinal microvasculature. The metabolic and ocular benefits of GLP-1RAs should be balanced with baseline ophthalmic evaluations, patient counseling on potential visual side effects, and close follow-up in clinical practice. Further research is warranted to focus on elucidating the mechanisms driving the contrasting effects of GLP-1RA on the eye and support evidence-based guidelines. Additionally, there is a need for further research on the true risk-benefit profile of GLP-1RA across ocular disease with adequately powered studies and controlled endpoints.
